# Hyperbilirubinemia as a Risk Factor for Mortality and Morbidity in Trauma Patients

**DOI:** 10.3390/jcm12134203

**Published:** 2023-06-21

**Authors:** Mina Lee, Myungjin Jang, Jayun Jo, Byungchul Yu, Giljae Lee, Jungnam Lee, Seunghwan Lee, Yangbin Jeon, Kangkook Choi

**Affiliations:** 1 Department of Traumatology, Gachon University, Incheon 21565, Republic of Korea; jwh@gilhospital.com (M.L.); saguri5919@gilhospital.com (M.J.); jayuncho@gilhospital.com (J.J.); kane2123@gilhospital.com (B.Y.); nonajugi@gilhospital.com (G.L.); jnlee@gilhospital.com (J.L.); surgeonrumi@outlook.kr (S.L.); junyb@gilhospital.com (Y.J.); 2 Department of Trauma Surgery, Gachon University Gil Medical Center, Incheon 21556, Republic of Korea

**Keywords:** trauma, hyperbilirubinemia, complication, mortality

## Abstract

Hyperbilirubinemia is frequently reported in trauma patients. However, few studies have investigated the effects of hyperbilirubinemia on patients’ clinical trajectories. This study aimed to evaluate the relationship between hyperbilirubinemia and patient outcomes following trauma. Our study included 387 patients who were admitted to the trauma bay with severe trauma between January 2017 and December 2021. We categorized patients into two groups based on their peak bilirubin levels: the low-bilirubin (LB) group, with levels below 3 mg/dL, and the high-bilirubin (HB) group, with levels above 3 mg/dL. We then compared the rates of complications and mortality between these two groups. The incidence of pneumonia (10.8% vs. 32.3%, *p* < 0.001), acute kidney injury (AKI) (2.8% vs. 19.2%, *p* < 0.001), sepsis (2.8% vs. 10.1%, *p* = 0.003), and wound infections (8.3% vs. 30.3%, *p* < 0.001) was significantly higher in the HB group. Additionally, the mortality rate was significantly higher (4.2% vs. 10.1%, *p* = 0.028) in the HB group. Multivariate analysis revealed that the higher the bilirubin level, the greater the risk of complications (pneumonia: odds ratio [OR] = 3.238; 95% confidence interval [CI] = 1.68–6.22; *p* < 0.001, AKI: OR = 4.718; 95% CI = 1.65–13.44; *p* = 0.004, sepsis: OR = 3.087; 95% CI = 1.00–9.52; *p* = 0.04, wound infection: OR = 3.995; 95% CI = 2.073–7.700; *p* < 0.001). In conclusion, hyperbilirubinemia was associated with poorer outcomes in trauma patients.

## 1. Introduction

Hyperbilirubinemia is frequently observed, occurring in as many as 45% of patients admitted to surgical intensive care units following severe trauma or major surgery [1,2,3,4]. The underlying causes of hyperbilirubinemia in trauma patients can be broadly divided into three primary mechanisms: prehepatic, intrahepatic, and posthepatic causes [5].

In critically ill patients, the causes of hyperbilirubinemia include biliary obstruction, liver disease, hemolysis, hematoma resorption, and drug toxicity [6]. Trauma patients, however, can develop hyperbilirubinemia even without pre-existing hepatobiliary disease. The main causes of jaundice in trauma patients are typically associated with factors such as initial shock, systemic hypotension, blood transfusion, and hematoma [7,8].

Several studies have reported that slightly elevated serum bilirubin levels within the normal range have beneficial effects on health due to bilirubin’s strong antioxidant, anti-inflammatory, immunomodulatory, and anti-excitotoxic properties [9,10]. However, toxicity paradoxically ensues when the concentration of total serum bilirubin surpasses a certain threshold, leading to oxidative stress, inflammation, apoptosis/necrosis, and excitotoxicity [11,12].

Bilirubin has been utilized as a measure of liver dysfunction in critically ill patients, serving as an indicator for liver function in the Acute Physiology and Chronic Health Evaluation (APACHE) score [13] and the Sequential Organ Failure Assessment (SOFA) score [14]. The SOFA score during the initial 24 h has proven to be an excellent prognostic marker for mortality in critically ill patients [15,16]. Several studies have reported that hyperbilirubinemia affected approximately 40% of critically ill patients and was associated with mortality and poor outcomes [17,18].

Studies have focused on the complex causes and mechanisms underlying hyperbilirubinemia in trauma patients [2,19,20]. However, there has been a lack of research investigating whether bilirubin can serve as an effective prognostic indicator in trauma patients, in whom hyperbilirubinemia may arise not only due to liver organ dysfunction but also due to multiple other factors.

In light of the existing literature, we hypothesized that hyperbilirubinemia increases the risk of complications and mortality in trauma patients. The purpose of this study was to evaluate the relationship between hyperbilirubinemia and outcomes in severe trauma patients.

## 2. Materials and Methods

### 2.1. Setting

This study was conducted at the trauma center of Gachon University Gil Hospital in Republic of Korea. Gachon University Gil Hospital, a teaching hospital with 1500 beds, is situated in Incheon and serves a population of 3 million people. Our hospital’s trauma center, the first of its kind in Republic of Korea, meets the standards of a level I trauma center. With over 3000 trauma-related admissions annually, including 500–550 cases of major trauma (Injury Severity Score [ISS] > 15), the center is well-equipped. It boasts a trauma bay, two operating rooms dedicated to trauma, a 20-bed trauma intensive care unit, and a trauma angiography suite exclusively for trauma patients. A hybrid operating room is also available for simultaneous endovascular intervention and open surgery. The center currently employs 14 full-time trauma surgeons, with a team of emergency physicians, anesthesiologists, and radiologist consultants available 24/7.

This study was approved by the local ethics committee (GCIRB2023-146).

### 2.2. Inclusion and Exclusion Criteria

The study included patients who visited the trauma bay with severe trauma between January 2017 and December 2021. During this period, 5508 patients were admitted to the trauma bay. We included patients who were admitted to the intensive care unit for more than 48 h due to a torso injury, two or more long bone fractures, a pelvic bone fracture, or combined injuries. Patients younger than 18 years of age were excluded. Patients with pre-existing conditions such as liver cirrhosis, alcoholic hepatitis, and cholecystitis were also excluded from the study.

### 2.3. Data Collection

Data on patients were retrospectively collected from electronic medical records. We gathered the following information: age, sex, physiological measurements upon ER admission (body temperature, systolic arterial blood pressure [SBP], heart rate, respiratory rate, and the Glasgow Coma Scale [GCS]), biochemical parameters (arterial pH, lactate, aspartate aminotransferase, alanine aminotransferase, total bilirubin), Acute Physiology and Chronic Health Evaluation II (APACHE II) score, ISS, Abbreviated Injury Scale (AIS), amount of transfusion within 24 h, complications (pneumonia, acute kidney injury [AKI], sepsis, wound infection), length of stay in the intensive care unit, total hospital stay, and mortality. Serum bilirubin levels were recorded during the patients’ hospital stay. These levels were measured on the day of admission and on days 2 and 7 of hospitalization. Peak bilirubin levels within the first 7 days were also examined. Patients were divided into two groups based on their peak bilirubin levels: a low-bilirubin (LB) group (<3 mg/dL) and a high-bilirubin (HB) group (>3 mg/dL). The cutoff level was chosen on the premise that a bilirubin level of 3 mg/dL would be visibly noticeable, thereby prompting a bilirubin level measurement for diagnosis. Several studies have identified a correlation between hyperbilirubinemia and infections in patients in the surgical intensive care unit. It was determined that patients with a blood bilirubin level of 3 mg/dL or higher are at an increased risk of infection, and this risk escalates with increasing bilirubin levels [2,4,21,22,23]. Additionally, based on a receiver operating characteristic (ROC) curve analysis, the cutoff values of serum bilirubin were 1.790, 3.195, 1.875, 1.990, and 2.340 for the outcomes of pneumonia (area under the curve [AUC] = 0.734, *p* < 0.001), AKI (AUC = 0.816, *p* < 0.001), sepsis (AUC = 0.757, *p* < 0.001), wound infection (AUC = 0.737, *p* < 0.001), and death (AUC = 0.683, *p* = 0.004), respectively. The cutoff value of 3 mg/dL serum bilirubin was used for analyses in this study because the cutoff value of serum bilirubin for AKI was 3.195, with a very high AUC (more than 0.8).

### 2.4. Statistical Analysis

Continuous data are expressed as means and standard deviations or as medians and interquartile ranges. Univariate analysis was conducted using the Student t-test for continuous variables and the chi-square test for categorical variables. Logistic stepwise regression analysis was used to predict the relationship between hyperbilirubinemia and patient outcomes. The threshold for statistical significance was set at *p* < 0.05. The odds ratios and 95% confidence intervals were calculated. All statistical analyses were performed using SPSS version 20.0 (IBM Corp., Armonk, NY, USA).

## 3. Results

During the study period, we included 387 patients. Based on the peak bilirubin level measured within 7 days after admission, we divided the patients into the HB group, with a serum bilirubin level of 3 mg/dL or more, and the LB group, with a serum bilirubin level of less than 3 mg/dL. The LB group consisted of 288 patients, while the HB group included 99 patients. 

We compared the demographic characteristics of these two groups. There were no significant differences in age or sex between the two groups. However, differences were noted in SBP (121.9 ± 36.4 vs. 103.7 ± 36.3, *p* < 0.001), lactate level (3.6 ± 2.8 vs. 4.9 ± 3.0, *p* < 0.001), and arterial pH (7.34 ± 0.18 vs. 7.29 ± 0.22, *p* = 0.008) upon admission. There was no significant difference between the two groups regarding liver injury (47 [16.3%] vs. 21 [21.2%] patients, *p* = 0.27). Significant differences were found in the volume of transfusion within the first 24 h (2.6 ± 3.8 vs. 6.8 ± 9.3 units, *p* < 0.001) and the ISS (19.8 ± 10.3 vs. 27.1 ± 10.0, *p* < 0.001) (Table 1).

We also compared the outcomes of the two groups. The HB group experienced a greater number of complications. Incidences of pneumonia (10.8% vs. 32.3%, *p* < 0.001), acute kidney injury (AKI) (2.8% vs. 19.2%, *p* < 0.001), sepsis (2.8% vs. 10.1%, *p* = 0.003), and wound infections (8.3% vs. 30.3%, *p* < 0.001) were significantly higher in the HB group. The durations of the intensive care unit (ICU) stay (7.3 ± 10.2 vs. 14.2 ± 16.0, *p* < 0.001) and overall hospital stay (21.9 ± 21.9 vs. 30.7 ± 23.4, *p* < 0.001) were also longer in the HB group. Mortality was significantly higher (4.2% vs. 10.1%, *p* = 0.028) in the HB group (Table 2).

An ROC curve analysis was conducted to evaluate the association between total bilirubin levels and individual comorbidities as well as outcomes. The serum bilirubin cutoff values for various outcomes were as follows: 1.790 for pneumonia (AUC = 0.734, *p* < 0.001), 3.195 for AKI (AUC = 0.816, *p* < 0.001), 1.875 for sepsis (AUC = 0.757, *p* < 0.001), 1.990 for wound infection (AUC = 0.737, *p* < 0.001), and 2.340 for death (AUC = 0.683, *p* = 0.004) (Table 3).

We used multivariate analysis with covariates to determine if there was a link between hyperbilirubinemia, complications, and mortality. The covariates were SBP, GCS, lactate, transfusion, and ISS, which all showed significant differences in earlier comparisons. The multivariate analysis results revealed that higher bilirubin levels were associated with an increased risk of pneumonia, AKI, sepsis, and wound infection. For pneumonia, the odds ratio (OR) was 3.238, with a 95% confidence interval (CI) of 1.68–6.22 (*p* < 0.001); for AKI, the OR was 4.718 (95% CI, 1.65–13.44; *p* = 0.004); for sepsis, the OR was 3.087 (95% CI, 1.00–9.52; *p* = 0.04); and for wound infection, the OR was 3.995 (95% CI, 2.073–7.700; *p* < 0.001). This multilevel analysis with covariates demonstrated that hyperbilirubinemia was a risk factor for complications. Higher bilirubin levels also correlated with increased mortality, though this trend was not statistically significant (Table 4).

**Figure 1:**
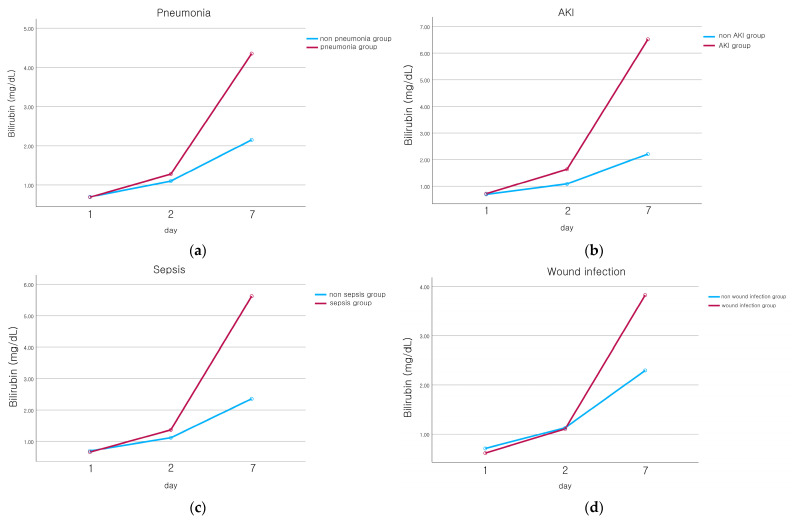
Serial bilirubin levels between the non-complicated group and the complicated group with regards to pneumonia, AKI (Acute kidney injury), sepsis, and wound infection. (**a**) Bilirubin level between the non-pneumonia group (blue line) and the pneumonia group (red line). In the pneumonia group, hyperbilirubinemia was significantly higher than in the non-pneumonia group on day 7 (*p* = 0.003). (**b**) Bilirubin level between the non-AKI group (blue line) and the AKI group (red line). In the AKI group, hyperbilirubinemia was significantly higher than in the non-AKI group on day 7 (*p* < 0.001). (**c**) Bilirubin level between the non-sepsis group (blue line) and the sepsis group (red line). In the sepsis group, hyperbilirubinemia was significantly higher than in the non-sepsis group on day 7 (*p* = 0.011). (**d**) Bilirubin level between the non-wound infection group (blue line) and the wound infection group (red line). In the wound infection group, hyperbilirubinemia was significantly higher than in the non-wound infection group on day 7 (*p* = 0.037).

**Figure 2:**
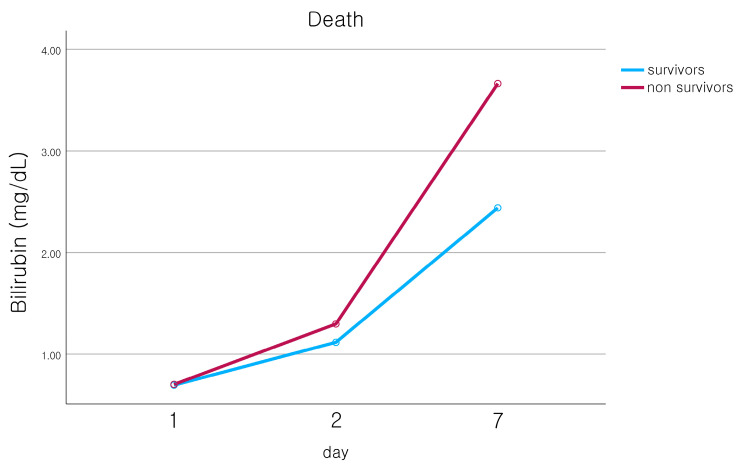
The time course of serial bilirubin levels between survivors (blue line) and non-survivors (red line). Respectively, hyperbilirubinemia developed in the non-survivors group, but there was no statistical significance (*p* = 0.325).

We collected serum bilirubin levels measured on days 1, 2, and 7 after hospitalization to see if there were any differences in the bilirubin level trends based on each complication. There was no significant difference in the initial bilirubin level at admission. However, in the pneumonia group, bilirubin levels rose over time, and this trend significantly differed from that of the non-pneumonia group. Similar patterns were observed in cases of AKI, sepsis, and wound infection, with all differences being statistically significant (Figure 1). The bilirubin levels also tended to increase more quickly in the group of patients who did not survive, though this increase was not statistically significant (Figure 2).

## 4. Discussion

This study revealed that hyperbilirubinemia is linked to increased complication rates and poor outcomes in trauma patients.

In patients with severe trauma, hyperbilirubinemia can be brought about by a multitude of factors. This condition could be the outcome of excessive bilirubin production, which may be triggered by blood transfusion, hematoma, blood leakage from damaged tissues, or hemolysis. Escalated endogenous production of bilirubin, resulting from the rapid breakdown of a large quantity of transfused erythrocytes during primary therapy, might be one of the contributing factors leading to progressive hyperbilirubinemia. In addition, hematoma absorption due to extravasation also contributes to bilirubin overload [24]. Hemorrhagic shock often induces metabolic disruptions in the liver [25,26,27]. The transportation of conjugated bilirubin from hepatocytes to the biliary tract must occur against a bilirubin concentration gradient that exists between the hepatocytes and the bile ducts. This energy-dependent process can be hindered by a disruption of hepatic energy metabolism in post-traumatic conditions, potentially also leading to bilirubinemia [28]. Liver dysfunction initiated by hemorrhagic shock can be further exacerbated by factors such as anesthesia, medications, and infections [3]. Hyperbilirubinemia due to trauma-induced injury or obstruction in the extrahepatic bile ducts is also possible, but it is less common than the mechanisms described above [29].

Multiple reports have identified the overproduction of bilirubin due to hemolysis of transfused blood and the degradation of extravascular blood as the leading causes of jaundice [29]. Our study also found significant differences in the amount of blood transfused in the early stages of treatment between the HB and LB groups. The ISS and lactate levels were noticeably different between the two groups, possibly due to extravasation and hematoma formation. Labori and colleagues noted that bleeding into soft tissues, fracture sites, and abdominal cavities is common in patients with severe trauma, leading to the necessity of extensive blood transfusions during primary therapy [30].

Blood transfusions provide patients with a large number of red blood cells, many of which have limited viability. These less viable red blood cells break down quickly. As this degradation progresses, the rate of endogenous bilirubin production in the body increases. Bilirubin can also be derived from the heme component of hemoglobin. Hemoglobin released from red blood cells during storage in blood banks provides an additional source for increased endogenous bilirubin production in transfused patients. The enhanced breakdown of transfused erythrocytes and extravasated blood can overwhelm the liver’s elimination capacity in trauma patients, leading to jaundice [3,4]. Some animal studies have reported that a large influx of bilirubin into the bloodstream can damage the tubular membrane, resulting in severe intrahepatic cholestasis [31]. These hepatocellular injuries and intrahepatic cholestasis can contribute to fatal outcomes if they develop in patients [32]. Labori et al. suggested that many patients with high ISS scores experience a disruption of the endogenous circulation of bile acids due to intestinal paralysis and can only maintain bile flow at a significantly reduced steady state. The liver becomes extremely vulnerable to the cholestatic effects of bilirubin overload under such circumstances. Other sources of endogenous bilirubin production include myoglobin and certain heme-containing enzymes. Therefore, muscle damage may also play a significant role in increasing endogenous bilirubin production in trauma patients [30].

Hepatic dysfunction is one of the causes of hyperbilirubinemia in trauma patients. Various studies have indicated that hepatic ischemia can occur due to shock or hypovolemia in the early stages of injury, even without a direct injury to the liver. In our study, there was no correlation between liver injury and hyperbilirubinemia. However, initial SBP and lactate levels were more severe in the HB group than in the LB group, and the ISS was higher in the HB group. This suggests that trauma patients in the HB group may have initially experienced shock or hypotension.

Several laboratory studies have provided evidence that hypotension can lead to liver dysfunction. Shoemaker et al. reported that the resistance to blood flow through the liver increased during shock [33], and Smith et al. found that portal vein flow decreased [34]. Brauer et al. suggested that hepatic excretion disorders occurred due to hepatic hypoperfusion [35]. Animal studies have shown that this damage persists even after recovery from initial hypotension. These findings support the hypothesis that post-traumatic liver dysfunction may be due to a period of liver ischemia in the early stages. Sarfeh et al. observed that the histologic findings in the liver of patients exhibiting hyperbilirubinemia after trauma were compatible with those seen in shock, specifically regarding the morphological manifestations of centrilobular congestion and necrosis [3]. Therefore, the correlation between electron microscopic findings in the liver and clinical symptoms of jaundice suggests that episodes of injury-induced shock may be a direct result of ischemic injury [1].

Several studies have examined the timing of hyperbilirubinemia’s onset after trauma. In our study, we monitored bilirubin levels for a week following admission. In the group with complications such as pneumonia, AKI, sepsis, and wound infection, bilirubin levels consistently rose, showing a significant difference compared to the group without complications by the seventh day. The patients who died also demonstrated a continuous increase in bilirubin, but there was no statistically significant difference compared to the control group. Several previous studies have reported that bilirubin levels typically peak between 6 and 12 days after trauma and have suggested that measurements of bilirubin levels during this period provide meaningful diagnostic and prognostic value [2,3,36,37].

In our study, we used a multivariable logistic regression model and found that high bilirubin levels were a risk factor for increased complications. Conditions such as pneumonia, AKI, sepsis, and wound infections occurred significantly more often in the HB group. While the mortality rate was also higher in the HB group, this difference was not statistically significant.

Previous studies have shown that elevated bilirubin levels are an independent prognostic factor for mortality in critically ill patients with sepsis from a diverse patient population [38,39]. As Zhai et al. noted, the prevailing consensus is that mortality rates increase in non-cirrhotic critically ill patients as bilirubin concentrations rise [40]. In a comprehensive multicenter study, Kramer et al. examined the mortality rates of critically ill non-cirrhotic patients in relation to bilirubin levels measured within the first 48 h of ICU admission. They observed a linear increase in the ORs for mortality, even at high bilirubin concentrations. However, they acknowledged potential differences in early and late liver dysfunction, and their study only evaluated hyperbilirubinemia present in the initial 48 h of admission [7]. Labori et al. reported that persistent hyperbilirubinemia exceeding 10–12 days after a severe injury is associated with the development of multiorgan failure and fatal outcomes in trauma patients [30].

Field et al. identified a correlation between hyperbilirubinemia and infections in the surgical intensive care unit. They found that patients with blood bilirubin concentrations of 3 mg/dL or higher were at an increased risk of infection, a risk that rose with higher bilirubin levels [21]. Hong et al. asserted that hyperbilirubinemia is significantly associated with mortality in critically ill patients, emphasizing the importance of assessing hyperbilirubinemia levels when predicting the prognosis of these patients [41]. Juschten et al. reported that early hyperbilirubinemia correlates with a high disease severity score, frequent sepsis, shocks, and multiple disorders, and is also associated with mortality [42]. Additionally, Fan et al. showed that hyperbilirubinemia at the time of ICU admission correlates with mortality in patients with acute respiratory distress syndrome [43].

Multiple studies have indicated that hyperbilirubinemia can encourage infection. Hayashi et al. demonstrated that the enzyme heme oxygenase-1 (HO-1), which is involved in bilirubin metabolism, reduced leukocyte adhesion in response to oxidative stress in rats [44]. Keshavan et al. found that bilirubin inhibited vascular cell adhesion molecule-1 (VCAM-1)-mediated leukocyte migration and the production of reactive oxygen species in vitro. This inhibition can lessen leukocyte adhesion and migration, leading to a diminished immune response and a heightened risk of infection [45]. Adin et al. reported that a slight increase in serum bilirubin could provide antioxidative and other beneficial effects [46]. However, they also pointed out that hyperbilirubinemia can have deleterious effects on organs and cause irreversible damage. Hyperbilirubinemia may also trigger harmful outcomes by activating pathways related to vascular endothelial dysfunction and programmed cell death [47,48].

This study has some limitations. First, it was a retrospective cohort study, which imposes certain constraints on the generalization of our findings. Second, we cannot rule out the possibility that certain medications may have influenced serum bilirubin levels. Nonetheless, a prior study indicated that potentially hepatotoxic drugs administered to ICU patients did not significantly alter serum bilirubin levels [49]. Third, there may exist other pre-existing conditions associated with hyperbilirubinemia and recovery that we did not include. As such, this study may not have evaluated certain hidden prognostic factors.

Previous studies have similarly concluded that hyperbilirubinemia is a risk factor for poor outcomes in severely ill patients, but there have not been reports of changes in ICU policies or protocols as a result. Since our study also found that hyperbilirubinemia following trauma is associated with infectious complications, additional studies are necessary to determine whether improved outcomes can be achieved through the implementation of enhanced ICU protocols.

To our knowledge, no previous large-scale study has investigated the association between hyperbilirubinemia in trauma patients and their outcomes. The present study highlights an increased risk of complications and mortality associated with higher bilirubin levels in this patient population. This article contributes to the understanding of hyperbilirubinemia as a potential prognostic marker in trauma patients, providing valuable insights for clinical practice and future research in the field.

## 5. Conclusions

In conclusion, hyperbilirubinemia is significantly associated with complications and mortality in trauma patients, thus indicating poor outcomes. This study underscores the importance of monitoring patients for hyperbilirubinemia during an ICU stay to predict prognosis in trauma patients.

## Figures and Tables

**Table 1:** Comparison of demographic and clinical characteristics between the low-bilirubin (LB) group and the high-bilirubin (HB) group.

	LB Group n = 288	HB Group n = 99	*p* Value
Age	49.9 ± 18.7	47.8 ± 17.7	0.166
Sex (male, %)	212 (73.6%)	81 (81.8%)	0.1
SBP in ED	121.9 ± 36.4	103.7 ± 36.3	<0.001
GCS in ED	12.8 ± 3.8	12.2 ± 4.2	0.107
Lactate in ED	3.6 ± 2.8	4.9 ± 3.0	<0.001
Arterial PH in ED	7.34 ± 0.18	7.29 ± 0.22	0.008
Transfusion pRBC < 4 h	1.8 ± 3.1	4.1 ± 5.2	<0.001
Transfusion pRBC < 24 h	2.6 ± 3.8	6.8 ± 9.3	<0.001
Transfusion pRBC in admission	5.6 ± 8.1	18.8 ± 20.6	<0.001
Liver injury	47 (16.3%)	21 (21.2%)	0.27
APACHE II	19.2 ± 10.5	20.7 ± 9.7	0.094
ISS	19.8 ± 10.3	27.1 ± 10.0	<0.001

LB, low bilirubin; HB, high bilirubin; SBP, systolic blood pressure; ED, emergency department; GCS, Glasgow Coma Scale; pRBC, packed red blood cells; APACHE II, Acute Physiology and Chronic Health Evaluation II; ISS, Injury Severity Score.

**Table 2:** Comparison of outcomes between the low-bilirubin (LB) group and the high-bilirubin (HB) group.

	LB Group n = 288	HB Group n = 99	*p* Value
Complication			
Pneumonia	31 (10.8%)	32 (32.3%)	<0.001
AKI	8 (2.8%)	19 (19.2%)	<0.001
Sepsis	8 (2.8%)	10 (10.1%)	0.003
Wound infection	24 (8.3%)	30 (30.3%)	<0.001
Outcome			
ICU stay (d)	7.3 ± 10.2	14.2 ± 16.0	<0.001
LOH (d)	21.9 ± 21.9	30.7 ± 23.4	<0.001
30-day mortality (%)	12 (4.2%)	10 (10.1%)	0.028

LB, low bilirubin; HB, high bilirubin; AKI, acute kidney injury; ICU, intensive care unit; LOH, length of hospital stay.

**Table 3:** Area under the ROC curve (AUC) of the association between total bilirubin levels and outcomes.

	AUC	95% CI	Bilirubin Cut-Off Value	*p*
Pneumonia	0.734	0.669–0.798	1.790	<0.001
AKI	0.816	0.728–0.904	3.195	<0.001
Sepsis	0.757	0.671–0.844	1.875	<0.001
Wound infection	0.737	0.668–0.806	1.990	<0.001
Death	0.683	0.572–0.794	2.340	0.004

ROC, receiver operating characteristic; CI, confidence intervals; AKI, acute kidney injury.

**Table 4:** Multivariate analysis of the association between bilirubin levels and outcomes.

	*p*	OR	95% CI	
			Upper	Lower
Pneumonia	<0.001	3.238	1.685	6.222
AKI	0.004	4.718	1.656	13.441
Sepsis	0.049	3.087	1.001	9.525
Wound infection	<0.001	3.995	2.073	7.700
Death	0.172	1.999	0.740	5.400

OR, odds ratio; CI, confidence intervals; AKI, acute kidney injury.

## Data Availability

The data presented in this study are available on request from the corresponding author.
